# The effect of an odd-one-out visual search task on attentional bias, body size adaptation, and body dissatisfaction

**DOI:** 10.1098/rsos.231817

**Published:** 2024-07-17

**Authors:** T. House, I. D. Stephen, K. R. Brooks, H. Bould, A. S. Attwood, I. S. Penton-Voak

**Affiliations:** ^1^ School of Psychological Sciences, Faculty of Medicine, Health and Human Sciences, Macquarie University, Sydney, New South Wales 2109, Australia; ^2^ Perception in Action Research Centre (PARC), Macquarie University, Sydney, New South Wales 2109, Australia; ^3^ Lifespan Health & Wellbeing Research Centre, Macquarie University, Sydney, New South Wales 2109, Australia; ^4^ School of Psychological Science, University of Bristol, Bristol BS8 1QU, UK; ^5^ Centre for Academic Mental Health, Population Health Sciences, Bristol Medical School, University of Bristol, Bristol BS8 1QU, UK; ^6^ Gloucestershire Health and Care NHS Foundation Trust, Centre for Academic Mental Health, University of Bristol, Bristol BS8 1QU, UK; ^7^ MRC Integrative Epidemiology Unit, Bristol Medical School, University of Bristol, Bristol BS8 1QU, UK; ^8^ Department of Psychology, Bournemouth University, Bournemouth, UK; ^9^ National Institute for Health Research Bristol Biomedical Research Centre, University Hospitals Bristol NHS Foundation Trust and University of Bristol, Bristol, UK

**Keywords:** attention training, attentional bias modification, visual search, body size adaptation, body dissatisfaction

## Abstract

Body image disturbance is a both a risk factor for, and a symptom of, many eating disorders and refers to the misperception of and dissatisfaction with one's own body. Women with high body dissatisfaction have been shown to direct more attention to low body mass index (BMI) bodies, which results in the overestimation of body size via body size adaptation. Therefore, attention may have a causal role in body image disturbance. We conducted a novel training visual search task with 142 young adult women who we trained to attend to either high or low BMI bodies. We assessed the effects of this training on attention to bodies of different sizes, body size adaptation, and body dissatisfaction. Women trained to attend to low BMI bodies decreased their perceptions of a ‘normal’ body size via adaptation from pre- to post-training (*p* < 0.001); however, women trained to attend to high BMI bodies showed no change in their perception of a ‘normal’ body size. We found no lasting effects of the training on attention to body size or body dissatisfaction; however, our visual search task showed poor internal consistency as a measure of attention. These findings indicate that attention to low BMI bodies may exacerbate body image disturbance in women. However, more reliable measures of attentional are required to confirm this finding.

## 1. Introduction

The term body image disturbance refers to a person's negative subjective experiences of their own body [1,2]. Body image disturbance is a complex multidimensional concept, consisting of two main constructs. The first is body size and shape misperception—a perceptual construct referring to a person's over- or underestimation of their body size [3]. The second—body dissatisfaction—is an attitudinal construct referring to a person's negative evaluation of their body [4]. Both are associated with eating pathology. For example, body dissatisfaction and the overestimation of body size are associated with anorexia nervosa [5] and bulimia nervosa [1]. Body dissatisfaction is also a risk factor for bulimia nervosa, binge eating disorder, purging disorder [6], later depressive episodes [7], and risky health behaviours [8], as well as dieting and negative affect [9]. For these reasons, body image disturbance is considered a serious public health concern [7,8,10].

Body image disturbance is associated with multiple cognitive biases pertaining to body-related stimuli, including attentional, memory, and judgment biases [11]. Cognitive behavioural theories of eating disorders propose that the relationship is likely bidirectional, in that body image disturbance leads to biased cognitive processing of body-related stimuli, which in turn exacerbates feelings of body dissatisfaction [12]. One particular cognitive bias that has received considerable interest is attentional bias to bodies of different sizes. Western media has traditionally promoted a low body mass index (BMI) body size as ideal for women [13–17], and people tend to rate low BMI female bodies as more attractive [18–20]. This body size preference is reflected in women's attentional biases, as women reporting high levels of body dissatisfaction tend to direct more gaze to low weight female bodies [21].

By paying more visual attention to smaller body sizes, women with high body dissatisfaction may be worsening their body image disturbance via visual adaptation—a perceptual bias caused by exposure to extreme stimuli [3,22]. When people observe low (high) BMI bodies they visually ‘adapt’ to these bodies, overestimating (underestimating) the size of subsequently presented body stimuli [23–28]. These ‘body size aftereffects' are typically measured by asking participants at pre- and post-adaptation to select the body size they perceive as ‘normal’. Participants who adapt to low (high) BMI bodies overestimate (underestimate) the size of the post-adaptation body stimuli, and thus select smaller (larger) ‘normal’ body sizes [29]. Correlational research shows that body size aftereffects are related to attentional bias, as people presented with high and low BMI body stimuli simultaneously visually adapt to the body size they spend more time fixating on [30]. Experimental research provides evidence for fixations affecting the magnitude and direction of body size aftereffects, because people presented with high and low body stimulus pairs visually adapt to the body size they are instructed to look toward [31]. Therefore, by directing attention to low BMI bodies in everyday life, women with high body dissatisfaction are more likely to adapt to those bodies, causing body size aftereffects.

Importantly, body size aftereffects can lead to misperceptions of one's own body size. Brooks *et al.* [24] adapted participants to low (high) BMI unfamiliar body stimuli and found participants proceeded to overestimate (underestimate) the size of their own body. Therefore, attentional bias to low BMI bodies may lead women to overestimate their own body size via body size adaptation [24,32]. Given the sociocultural pressures for women to be thin [33], the overestimation of body size may also lead to increased feelings of body dissatisfaction. Correlational research indicates the overestimation of body size is positively associated with body dissatisfaction [34–36]. Further, some research shows body size aftereffects co-occur with changes in body dissatisfaction. Bould *et al.* [37] found that women exposed to high BMI bodies proceeded to underestimate the size of subsequently presented body stimuli and report feeling more satisfied with their own body. Indeed, experimental studies show that simple exposure to low BMI bodies leads to an immediate increase in state body dissatisfaction in women, even without prompts that would promote body-relevant rumination or cognition [38–40]. Therefore, women with high body dissatisfaction may be worsening their body image disturbance by directing attention and visually adapting to low BMI bodies.

Given the potential negative outcomes of attentional bias to low BMI bodies, a promising intervention for the treatment of body image disturbance is computer-based attention training (sometimes referred to as attentional bias modification [41,42]). Computer-based attention training has been found to be effective in other areas of mental health, for example, by shifting attention away from threatening stimuli and reducing symptoms of anxiety [43,44], albeit producing small effect sizes [45]. Given that simple exposure to low BMI bodies can cause an immediate increase in state body dissatisfaction in women [38–40], it is possible that training women to direct attention towards bodies of different sizes may cause immediate changes in body dissatisfaction. The interventions are relatively low in cost and intensity and so, if effective at reducing body image disturbance, they could provide a cost-effective adjunct to traditional talking therapies [41,42].

A number of studies have used attention training to modify body dissatisfaction (for a review, see [46]). Most of these studies used the training dot probe task to train attention. The training dot probe task involves briefly presenting participants with a stimulus pair consisting of a target and control stimulus, followed by a probe that participants respond to as quickly as possible. To train participants to attend to target stimuli, the probe replaces the target stimulus consistently over repeated trials [47]. House *et al.* [48] used the training dot probe task to direct participants' attention to high versus low BMI bodies and found the training had no effect on participants’ body dissatisfaction and did not induce body size aftereffects. However, in the majority of cases this paradigm also failed to modify attention, which was assessed using reaction times on an assessment version of the dot probe task. Therefore, the absence of change for the body image variables is unsurprising. Other studies using the training dot probe task to direct attention to other body-related stimuli (e.g. body-related words) have similarly found minimal effects of attention training on body dissatisfaction [46,49]. However, many of these studies did not assess the effects of attention training on attentional bias, and therefore it is difficult to determine whether we would expect to see changes in body dissatisfaction. Studies that did assess attentional bias typically did so using reaction times on an assessment version of the dot probe task. However, the assessment version of the dot probe task has notably poor reliability [50–54] and, unlike eye-tracking measures, does not reliably detect positive associations between body dissatisfaction and attentional bias [21]. Therefore, the assessment dot probe task may not be an appropriate method of evaluating the effectiveness of attention training tasks.

An alternative less commonly used method of attention training is the training visual search task, which involves participants searching for a target stimulus amongst distractor stimuli. Over repeated training visual search trials, participants gradually become quicker at detecting target stimuli, reflecting increased attentional processing of those targets (and other stimuli paired visually adjacent to them [55]). Training visual search tasks have been successful at inducing immediate changes in body dissatisfaction by increasing attention to socially accepting versus threatening faces [56] and to attractive versus unattractive body parts [57]; therefore, they may present a more effective method of attention training than training dot probe tasks. Visual search tasks also tend to produce more reliable estimates of attentional bias than dot probe tasks [58,59]; therefore, they could provide a more reliable assessment of whether training visual search tasks are effective at modifying attentional bias.

In the present study, we aimed to investigate whether a novel training visual search task could be used to modify women's attention to high versus low BMI female bodies. Half of the participants trained to attend to high BMI body stimuli and half trained to attend to low BMI body stimuli. Participants were measured at pre- and post-training on their attentional bias to body size, body size perception, and body dissatisfaction. We hypothesized that participants trained to attend to low (high) BMI body stimuli would (1) increase attention to low (high) BMI body stimuli, (2) perceive lower (higher) BMI body stimuli as ‘normal’ due to body size adaptation, and (3) exhibit higher (lower) body dissatisfaction. This experiment was preregistered on the Open Science Framework (https://doi.org/10.17605/OSF.IO/NF8JX) with minor deviations from the preregistration explained in the electronic supplementary material (instead of ANCOVAs we conducted ANOVAs and used linear regressions on the post-training scores to check the effect of controlling for BMI).

## 2. Methods

### 2.1. Recruitment and participants

Participants were recruited using the University of Bristol's Experimental Hours Scheme and reimbursed with course credit. To take part in the experiment, participants had to identify as female, aged 18–35 years old, fluent in English, and as having normal or corrected-to-normal vision. The experiment was completed online and required computer keyboard responses, so we excluded participants if they used a phone or tablet device. A power analysis indicated a sample size of 142 participants would be sufficient to detect a small–medium interaction (time×attention training condition) using a 2 × 2 ANOVA with 80% statistical power and an alpha level of 5% (G*Power v3.1.9.2 [60]). Therefore, we aimed to recruit 142 participants. If a participant completed the study but failed our data screening checks (described in the Data analysis section), then we excluded the participant and recruited a replacement participant.

### 2.2. Stimuli

Body stimuli were obtained from the complete Morphed Photographic Figure Scale (MPFS) [61] set, which consists of photographs of ten women (mean age = 21.90 years, SD = 4.43; mean BMI = 19.64 kg m^−2^, SD = 2.74) who consented to their photographs being used in future research. To create the original MPFS set, Skinner and colleagues altered the apparent BMI of the identities in the ten photographs using PsychoMorph [62]. BMI transforms were based on the shape, colour, and texture differences between templates of averaged photographs of high BMI women (mean BMI: 25.2 kg m^−2^) and averaged photographs of low BMI women (mean BMI: 17.3 kg m^−2^). Skinner and colleagues transformed each of the ten original photographs to produce a sequence of nine morph levels from each photograph. Each sequence of nine morph levels included the original photograph plus four morph levels with the identity gradually increasing in apparent BMI and four morph levels with the identity gradually decreasing in apparent BMI (±20, ± 40, ± 60, and ±80% of the shape, colour, and texture differences between the low and high BMI templates).

**Figure 1.**
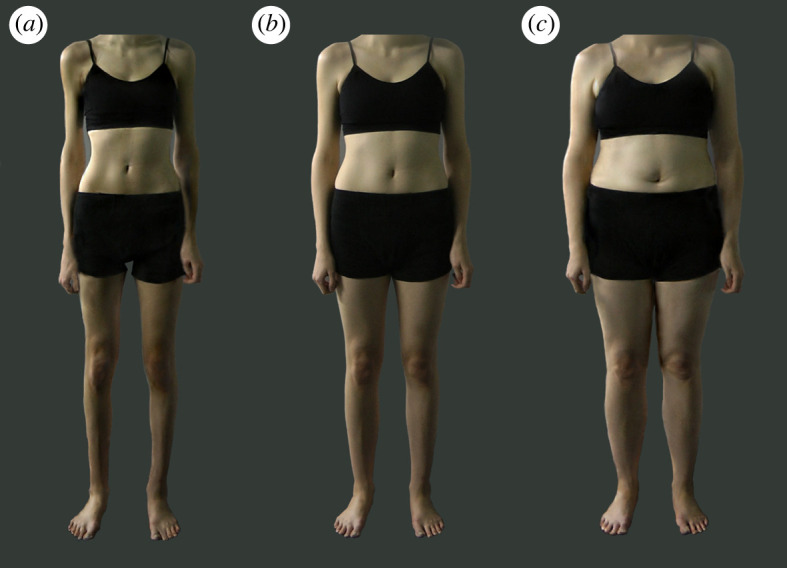
Example body stimuli depicting the same identity at varying degrees of apparent body mass index (BMI). (*a*) The low BMI version of the identity (i.e. the smallest transformed morph level), (*b*) the average BMI version of the identity (i.e. the unmanipulated morph level), and (*c*) the high BMI version of the identity (i.e. the largest transformed morph level).

To increase the sensitivity of the scale for the present experiment, we completed additional shape, colour, and texture transforms on the scale set using the same landmark points in PsychoMorph as applied by Skinner and colleagues [61]. We added an additional four steps to each sequence, resulting in 13 morph levels for each of the ten sequences. Each new sequence of 13 morph levels involved the original photograph plus six versions with the identity gradually increasing in apparent BMI and six versions with the identity gradually decreasing in apparent BMI (±13.3, ± 26.7, ± 40.0, ± 53.3, ± 66.7, and ±80.0% of the shape, colour, and texture differences between the averaged low and high BMI photographs). We then used the GIMP image editor platform (version 2.10.22) to add a grey background to each image and a grey layer to cover the identity's face (hexadecimal colour = #333935; figure 1). The size of the body stimuli varied based on the screen size of the participant's device, but the aspect ratios were identical for all participants.

### 2.3. Training visual search task

Participants’ attention was trained using a novel training version of a compound visual search task [63,64]. The task was completed on a computer and consisted of 360 trials presented in 6 blocks of 60 trials with a 30 s break between each block to reduce fatigue. Each trial started with a fixation cross presented for 1000 ms in the centre of the screen. The fixation cross then disappeared, and eight body stimuli appeared on the screen in front of a grey background (hexadecimal colour = #333935). The bodies were positioned with their centres evenly spaced in a circular array which was centred in the middle of the screen with a radius that was 34% of the screen's height. The dimensions of each body were 22% of the screen's height and 6% of the screen's width. The eight body stimuli involved one identity selected at random from the pool of ten. For participants trained to attend to high (low) BMI body stimuli, each trial displayed seven average BMI body stimuli and one high (low) BMI body stimulus. The average BMI body stimuli were always the unmanipulated version of the identity, and the high (low) BMI body stimulus was always the largest (smallest) transformed version. The position of the high or low BMI body stimulus in the circular array was randomized for each trial.

**Figure 2.**
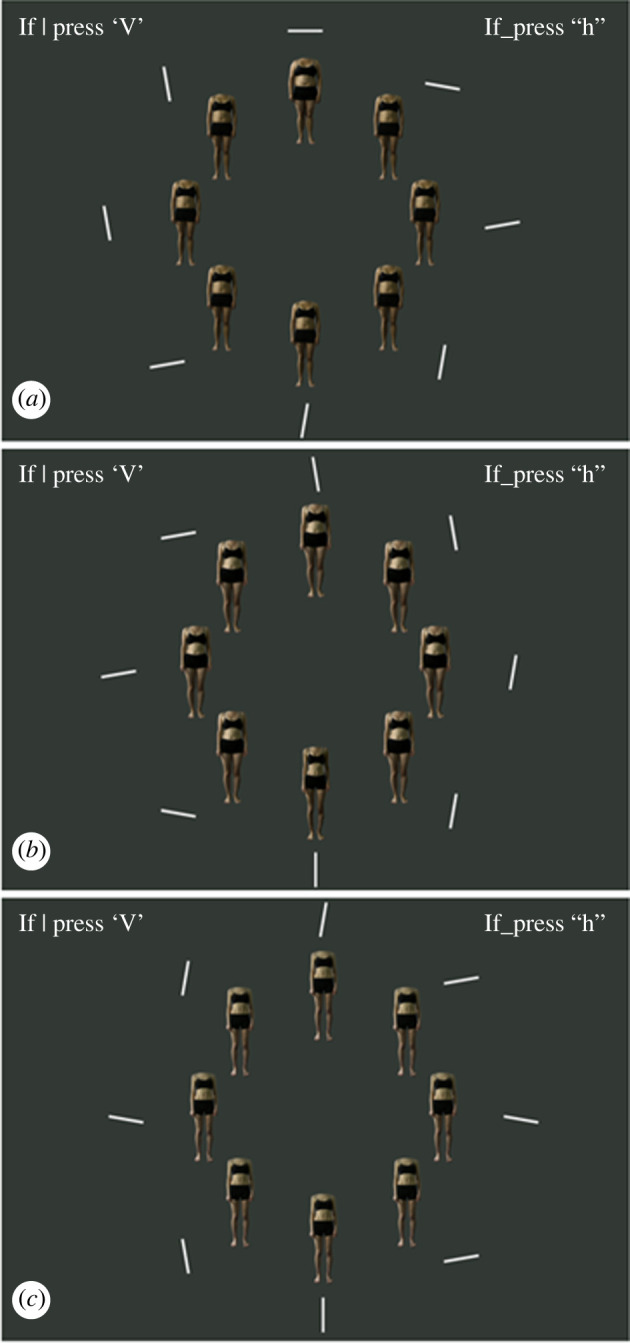
Example visual search trials. (*a*) An example training/pre-training target visual search trial for participants trained to attend to high body mass index (BMI) body stimuli. In this example, the target bar is the horizontal bar at the top centre of the array. The target bar is paired adjacent to a high BMI body stimulus and the remaining body stimuli are average BMI. (*b*) An example training/pre-training target visual search trial for participants trained to attend to low BMI body stimuli. In this example, the target bar is the vertical bar at the bottom centre of the array. The target bar is paired adjacent to a low BMI body stimulus and the remaining body stimuli are average BMI. (*c*) An example pre-training neutral visual search trial. The target bar is vertical at the bottom centre of the array and all body stimuli are average BMI.

Each body stimulus was paired with a short white bar (hexadecimal colour = #FFFFFF) that was located immediately adjacent to the body stimulus and presented simultaneously. The centres of the bars were evenly spaced in a larger circular array centred in the middle of the screen with a radius that was 44% of the screen's height. The dimensions of each bar were 6% of the screen's height and 1% of the screen's width. For each trial, one ‘target’ bar was randomly orientated at either a horizontal or vertical angle. The remaining seven ‘distractor’ bars were randomly oriented at either 80°, 100°, 170°, or 190°. For each trial, participants were required to indicate, as quickly as possible, whether a horizontal or vertical bar was present by pressing the appropriate keys (‘h’ or ‘v’). For participants trained to attend to high (low) BMI body stimuli, the target bar was paired with the high (low) BMI body stimulus on 100% of the trials. The seven distractor bars were paired at random with the remaining seven average BMI body stimuli. For each trial, the visual search display remained on the screen until the participant responded, whereupon they automatically proceeded to the next trial (figure 2).

### 2.4. Measures

#### 2.4.1. Attentional bias

To check whether the attention manipulation was successful, all participants completed pre- and post-training assessment versions of the visual search task that were designed to measure, rather than train, attentional bias [63,64]. Participants were measured on their change in attentional bias to the body size targeted in their attention training. We chose not to measure participant's change in attentional bias to other body sizes to minimize the effects of participant fatigue on data quality. The pre- and post-training visual search tasks each involved one block of 40 target trials and one block of 40 neutral trials. The order of the blocks was randomized for each participant, with no breaks. Target trials were identical to the participants' training visual search trials. Neutral trials were similar to target trials; however, participants were presented with eight average BMI body stimuli with no high or low BMI body stimulus present. For these neutral trials, the target bar and seven distractor bars were paired at random with each of the eight average BMI body stimuli. For both target and neutral trials, the instructions were identical to those for the training visual search trials (figure 2).

To calculate a pre- and post-training attentional bias score for each participant, we initially screened the data at a trial level using preregistered criteria developed in similar research [65]. We excluded visual search trials if the participant responded incorrectly (4.31% of pre-training visual search trials; 3.89% of post-training visual search trials) or if the participant's reaction time was less than 200 ms (0.55% of correct pre-training visual search trials; 1.74% of correct post-training visual search trials) or more than 2.5 standard deviations above their mean reaction time (2.62% of correct pre-training visual search trials; 2.40% of correct post-training visual search trials). After screening, pre-training attentional bias scores were calculated by subtracting mean response times for pre-training target trials from the mean response times for pre-training neutral trials. The same was done to calculate participants' post-training attentional bias scores, i.e. mean response times for post-training target trials were subtracted from the mean response times for post-training neutral trials. For participants trained to attend to high (low) BMI body stimuli, a positive attentional bias score meant that participants demonstrated an attentional bias to high (low) BMI body stimuli, relative to average BMI body stimuli.

#### 2.4.2. Point of subjective normality

To measure body size perception, and potentially detect an adaptation aftereffect, participants’ points of subjective normality (PSNs) were obtained at pre- and post-training with a version of the method of adjustment task [66]. Participants were presented with the ten body stimulus sequences one at a time in a random order. Participants were initially presented at random with one of the 13 morph levels of a single identity presented at the centre of screen. The body dimensions were 57% of the screen's height and 15% of the screen's width. Participants could manipulate the identity's body size by pressing ‘p’ on the keyboard to move to the next highest morph level and pressing ‘q’ on the keyboard to move to the next lowest morph level. The sequence of morph levels was looped, so pressing ‘p’ on the highest morph level would lead to the lowest morph level, and vice versa. Participants were instructed to press a ‘Select’ button to choose the morph level of the body that they thought looked the most ‘normal’ sized. Participants were given the freedom to interpret the meaning of ‘normal’ sized since we did not provide them with a specific definition. Pressing the ‘Select’ button moved the participant onto the next identity, repeating the process until they selected a ‘normal’ body size for each of the ten identities. The mean body size chosen as ‘normal’ for the ten identities was calculated to determine each participant's PSN score. Therefore, a higher (lower) PSN score indicated the participant perceived bodies higher (lower) in BMI to be ‘normal’ in size. We interpreted a PSN increase (decrease) from pre- to post-training as evidence of body size aftereffects, because underestimating (overestimating) the size of post-training body stimuli would lead participants to select post-training bodies higher (lower) in BMI as ‘normal’ sized.

#### 2.4.3. Body dissatisfaction

Body dissatisfaction was measured at pre- and post-training using a modified version of the body shape satisfaction scale [48,67], which asked participants to rate their satisfaction with 18 parts or features of their body. Participants were asked to respond based on their feelings ‘at this moment’ to increase the likelihood of detecting changes in state body dissatisfaction caused by the training visual search task [68]. Participants responded using a slider scale rather than a Likert scale to minimize the likelihood that they would remember and reproduce their pre-training responses when completing the post-training scale. Response options for each of the 18 items ranged from 0 to 100 (0 as ‘Very satisfied’ and 100 as ‘Very dissatisfied’). A body dissatisfaction score was calculated by summing the responses for all 18 items; therefore, a higher score indicated greater body dissatisfaction. For participants trained to attend to high BMI bodies, Cronbach's alpha indicated excellent internal consistency at pre-training (*α* = 0.91) and post-training (*α* = 0.94). For participants training to attend to low BMI bodies, Cronbach's alpha indicated excellent internal consistency at pre-training (*α* = 0.92) and post-training (*α* = 0.95).

#### 2.4.4. Attention check

To screen for participants who were paying sufficient attention to the experiment instructions, we included two attention check questions. The pre-training attention check question asked ‘Based on the above text, what is 5 + 5?’, with the above text instructing participants to answer with the number ‘50’. The post-training attention check question asked, ‘Based on the text below, what is today's date?’, with the below text instructing participants to answer with the word ‘today’. Participants were able to complete the experiment and be fully reimbursed regardless of their responses to these questions. However, we only included participants in our data analysis if they respond correctly to both questions (i.e. Q1 ‘50’ and Q2 ‘today’).

### 2.5. Procedure

The study was hosted on the Gorilla Experiment Builder [69] (https://gorilla.sc/) and participants accessed the experiment via a hyperlink. First, they were asked to complete a consent form and confirm whether they met the eligibility criteria. Participants who did were then asked to complete a demographics questionnaire which asked their age, ethnicity, and whether they had a current or previous diagnosis of an eating disorder. Participants also provided their height and weight so that we could calculate their BMI (kg m^−2^). Participants then completed the pre-training body shape satisfaction scale, followed by the pre-training attention check question. Participants then completed three practice PSN trials, which involved three identities selected at random from the pool of ten identities, followed by the pre-training PSN task. Participants then completed 10 practice visual search trials, which were similar to the neutral trials in the pre- and post-training visual search tasks; however, participants were presented with a tick for responding correctly and a cross for responding incorrectly. Once the participant completed the 10 practice visual search trials, they were given the opportunity to reread the task instructions and repeat the practice visual search trials. If the participant did not want to revisit the task instructions or practice visual search trials, then they completed the pre-training visual search task, followed by the training visual search task, followed by the post-training tasks in the following order: body shape satisfaction scale, attention check question, PSNs, visual search task.

### 2.6. Data analysis

Data were initially screened at a participant level (for screening results, see electronic supplementary material). Participants were excluded from the analysis if they terminated the experiment before completion, responded incorrectly to either attention check question, or if their response accuracy was less than 80% on either the pre- or post-training visual search tasks. To minimize any effects of training decay on the post-training measures, we excluded participants from the analysis if they took longer than 90 min to complete the experiment or took longer than 60 min and were inactive (i.e. did not make any keyboard or mouse response) for longer than 5 min during the training visual search task or post-training measures. We evaluated the internal consistency of the pre- and post-training visual search tasks as measures of attentional bias using the splithalf R package [70], which estimates split half reliability statistics for cognitive tasks. We calculated the average Spearman–Brown corrected correlation coefficients for 5000 random splits of reaction time difference scores for target versus neutral visual search trials, separately by attention training condition (high versus low BMI) and time (pre-training versus post-training).

To test our three hypotheses, we conducted three 2 × 2 ANOVAs. The data satisfied ANOVA assumptions of linearity, normality, and homogeneity of variances. For each ANOVA, we included attention training condition as the between-participants independent variable (high versus low BMI) and time as the within-participants independent variable (pre-training versus post-training). The dependent variable for the first ANOVA was attentional bias score. The purpose of this ANOVA was to check whether the attention training worked at manipulating attention to the target body size. Hypothesis 1 would be supported if there was evidence for a main effect of time and participants in both conditions demonstrated a higher attentional bias score at post-training than pre-training. The dependent variable for the second ANOVA was PSN score. Hypothesis 2 would be supported if there was evidence for an interaction and participants in the low (high) BMI training group demonstrated a PSN score decrease (increase) from pre- to post-training. The dependent variable for the third ANOVA was body dissatisfaction score. Hypothesis 3 would be supported if there was evidence for an interaction and participants in the low (high) BMI training group demonstrated a body dissatisfaction increase (decrease) from pre- to post-training. For each ANOVA, we used a standard *p* < 0.05 criterion to evaluate effects and interactions, and we followed up interactions using *t*-tests.

We conducted two sensitivity analyses to assess the robustness of our main results by rerunning our main analyses but with certain participants removed from the sample. First, we reran the main analyses but excluded participants who confirmed in the demographics questionnaire that they had a current or previous diagnosis of an eating disorder. Second, we reran the main analyses but excluding extreme outlier participants who were more than three times the interquartile range outside the 25th and 75th percentiles for any of the dependent variables (attentional bias score, PSN score, and body dissatisfaction score). Finally, we conducted exploratory correlation tests (Pearson's *r*) on the association between pre-training scores (attention bias, PSN, and body dissatisfaction) separated by condition.

## 3. Results

The final sample consisted of 71 participants trained to attend to high BMI bodies (mean age = 19.62 years, SD = 1.63; mean BMI = 22.18 kg m^−2^, SD = 3.56) and 71 participants trained to attend to low BMI bodies (mean age = 19.39 years, SD = 1.70; mean BMI = 21.65 kg m^−2^, SD = 2.74). The majority of the participants (*n* = 121) identified as White/White British/White European/White American, 7 identified as Asian/Asian British, 3 as Black/African/Caribbean/Black British, 3 identified as Middle Eastern, and the remaining 8 as mixed/multiple ethnic groups. Ten participants confirmed they had a history of an eating disorder. The internal consistency of the visual search task was variable. For participants trained to attend to high BMI bodies, it demonstrated poor internal consistency at pre-training (Spearman–Brown coefficient = 0.46 [95% CI = 0.20, 0.64]) and moderate internal consistency at post-training (Spearman–Brown coefficient = 0.71 [95% CI = 0.57, 0.82]). For participants trained to attend to low BMI bodies, internal consistency was poor at pre-training (Spearman–Brown coefficient = 0.17 [95% CI = −0.15, 0.45]) and post-training (Spearman–Brown coefficient = 0.49 [95% CI = 0.29, 0.65]).

**Figure 3.**
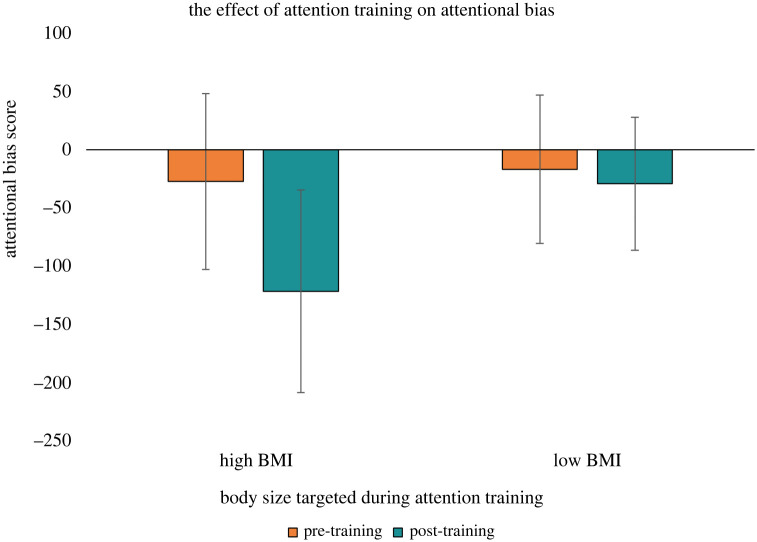
A bar chart depicting the effect of the attention training on the participants' attentional bias score (*N* = 142). For participants trained to attend to high (low) BMI body stimuli, a positive attentional bias score meant that participants demonstrated an attentional bias to high (low) BMI body stimuli, relative to average BMI body stimuli. Note: error bars indicate 95% confidence intervals.

**Figure 4.**
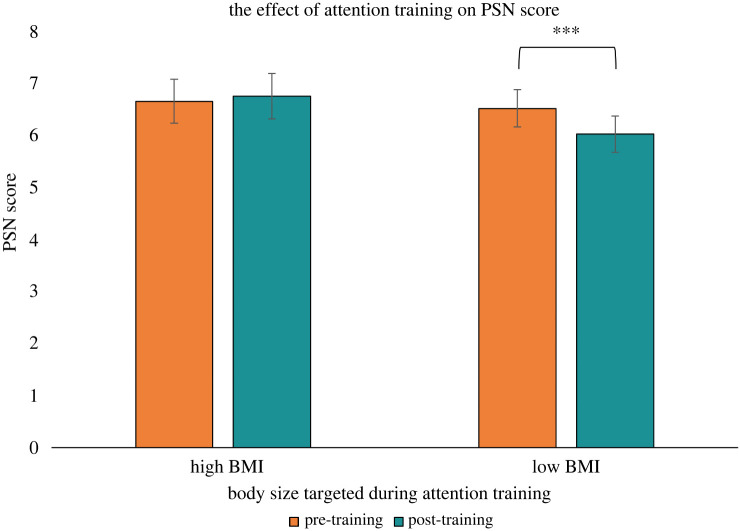
A bar chart depicting the effect of the attention training on the participants’ PSN score (*N* = 142). A higher (lower) PSN score indicated the participant perceived bodies higher (lower) in BMI to be ‘normal’ in size. Note: error bars indicate 95% confidence intervals. ***p < 0.001.

**Figure 5.**
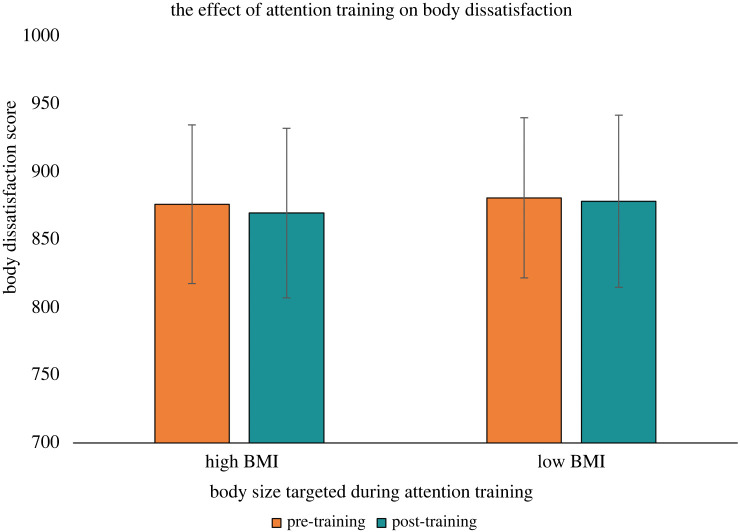
A bar chart depicting the effect of the attention training on the participants' body dissatisfaction score (*N* = 142). A higher body dissatisfaction score indicated greater body dissatisfaction. Note: error bars indicate 95% confidence intervals.

Descriptive statistics for attentional bias, PSN, and body dissatisfaction scores are reported in figures 3–5, with mean response times by trial type on the visual search task reported in table 1. The ANOVA for attentional bias score did not provide evidence for main effects of time, *F*_1,140_ = 2.25, *p* = 0.136, $\eta_{G}^{2}=0.007,$ condition, *F*_1,140_ = 1.86, *p* = 0.175, $\eta_{G}^{2}=0.007,$ or an interaction between time and condition, *F*_1,140_ = 1.33, *p* = 0.251, $\eta_{G}^{2}=0.004.$ The second ANOVA had PSN score as the outcome and provided evidence for a main effect of time, *F*_1,140_ = 6.31, *p* = 0.013, $\eta_{G}^{2}=0.004,$ no evidence for an effect of condition, *F*_1,140_ = 2.54, *p* = 0.113, $\eta_{G}^{2}=0.016,$ and strong evidence for an interaction between time and condition, *F*_1,140_ = 13.66, *p* < 0.001, $\eta_{G}^{2}=0.008.$ Follow up paired *t*-tests showed there was no evidence for a difference between pre- and post-training PSN scores for the high BMI attention training condition *t*_70_ = −0.84, *p* = 0.406, *d* = −0.099. For the low BMI training condition, there was strong evidence indicating participants decreased their PSN score from pre- to post-training, *t*_70_ = 4.40, *p* < 0.001, *d* = 0.522. An exploratory independent *t*-test on the post-training PSN scores showed evidence that participants in the low BMI training condition had a lower post-training PSN score compared to participants in the high BMI training condition, *t*_140_ = 2.56, *p* = 0.011, *d* = 0.430. The third ANOVA had body dissatisfaction score as the outcome and did not provide evidence for a main effect of time, *F*_1,140_ = 0.79, *p* = 0.376, $\eta_{G}^{2}=0.001,$ condition, *F*_1,140_ = 0.02, *p* = 0.878, $\eta_{G}^{2}=0.001,$ or an interaction between time and condition, *F*_1,140_ = 0.16, *p* = 0.694, $\eta_{G}^{2}=0.001.$ The sensitivity analyses produced consistent results to our main analyses (see electronic supplementary material), indicating the results were not driven by extreme outlier participants or those with an eating disorder history. The results of the correlation analysis are reported in table 2 and do not provide evidence for associations at pre-training between attentional bias score, PSN score, or body dissatisfaction score (for all tests: *p* > 0.05 and −0.2 < *r* < 0.2).

**Table 1.** The mean response times for the neutral and target trials, reported separately for attention training condition and pre- and post-training (standard deviation reported in parentheses).

attention training condition	pre-training		post-training	
	neutral	target	neutral	target
high BMI	2165.96 (421.93)	2193.09 (474.18)	1735.15 (423.56)	1856.36 (570.39)
low BMI	2251.82 (480.20)	2268.62 (451.55)	1727.50 (309.14)	1756.56 (314.69)

**Table 2.** The results of the correlation analysis on pre-training scores, separated by condition (*N* = 71 per condition; d.f. = 69 per test).

	attentional bias		PSN	
	*r*	*p*	*r*	*p*
high BMI attention training condition				
attentional bias	—	—	—	—
PSN	−0.16	0.191	—	—
body dissatisfaction	0.02	0.898	−0.11	0.345
low BMI attention training condition				
attentional bias	—	—	—	—
PSN	−0.14	0.243	—	—
body dissatisfaction	−0.19	0.106	0.06	0.621

## 4. Discussion

We used a novel training visual search task to train the attention of young adult women to either high or low BMI female bodies. In support of our second hypothesis, participants trained to attend to low BMI bodies showed a visual aftereffect of size overestimation, as demonstrated by a decrease in the size of bodies deemed to appear ‘normal’ from pre- to post-training. Contrary to our first and third hypotheses, there was no evidence suggesting participants trained to attend to low BMI bodies changed their attentional bias or body dissatisfaction from pre- to post-training. Contrary to all three hypotheses, there was no evidence suggesting participants trained to attend to high BMI bodies changed their attentional bias, perception of body size, or body dissatisfaction from pre- to post-training.

The participants trained to attend to low BMI bodies adapted to low BMI bodies without demonstrating a lasting measurable change in attention from pre- to post-training. Therefore, participants in this condition may have adapted to low BMI bodies simply via increased exposure to low BMI bodies during the training visual search trials. This interpretation is consistent with the research showing that exposure to low BMI bodies leads participants to overestimate the size of subsequently presented body stimuli [24,25]. Adaptation can transfer across identities and can lead to the misperception of one's own body size [24,32]; therefore, these results support the suggestion that increased exposure to low BMI bodies could lead a person to overestimate their own body size [3,22]. The overestimation of body size is a core feature and diagnostic symptom of anorexia nervosa [5,71]; therefore, exposure induced body size adaptation may be a contributing factor in the development and/or maintenance of eating disorders.

Participants adapted to low BMI bodies; however, they did not report an increase in body dissatisfaction following the attention training. This lack of change in body dissatisfaction is inconsistent with some previous research that found body size aftereffects co-occurred with changes in body dissatisfaction [37]. On the other hand, Stephen and colleagues [31] found that increased attention to low BMI bodies led to body size aftereffects but no changes in body dissatisfaction. One possible explanation for the inconsistent findings is that Bould and colleagues [37] asked participants to look in a full length mirror immediately after the adaptation period, which could have distorted the participants' stored representation of their body size [29] and increased translation effects on body dissatisfaction. However, simple exposure to low BMI bodies can cause an immediate increase in state body dissatisfaction in women, even without seeing their own body or having the opportunity for body-related rumination [38–40]; therefore further research is needed to test this explanation. Another possible explanation is that the effects of low BMI bodies on body dissatisfaction are diluted when other body sizes are presented simultaneously, as was the case in the present study and the study conducted by Stephen and colleagues [31].

We did not find evidence that the attention training had a lasting effect on attention to high versus low BMI bodies. Although we expected faster responses to the target body size in post- compared to pre-training assessments, we did not find any such change. The low BMI attention training condition involved directing participants’ attention to low BMI bodies and participants in this condition did show a body size aftereffect. Therefore, it is possible that these participants increased their attention to low BMI bodies even though we did not detect a change in reaction times. We evaluated the reliability of the visual search task as a measure of attentional bias and found the task had poor to moderate internal consistency. These results are more promising than results from dot probe tasks (e.g. [50]); however, they are still unacceptably low. It is not yet standard practice to report on the psychometric properties of cognitive behavioural tasks [51]; therefore, it is difficult to compare the internal consistency of our version of the visual search task to previous versions [63,64]. However, the results suggest the visual search task may not have been sufficient for detecting measurable changes in attention. This suggestion is consistent with the lack of evidence from the dot probe task and visual task for an association between body dissatisfaction and attentional bias to low BMI bodies, which contradicts a recent meta-analysis which found evidence from eye-tracking studies for a positive association [21,50].

Given participants in the low BMI attention training condition did adapt to low BMI bodies, the training visual search task may have promise as a method of attention training (albeit to low BMI bodies which were not our target for therapeutic intervention). Future research assessing the effects of attention training on attention should consider using alternative measures of attention. For example, although more costly and resource intensive, eye-tracking measures such as total dwell time have good to excellent internal consistency results [72,73]. Similarly, event-related potentials (ERPs) produce excellent internal consistency results [74] and are reliably modified by attention training tasks [75]. In addition, a control condition should be included to distinguish between aftereffects caused by exposure versus attention to low BMI bodies by training participants to attend to average sized bodies in the presence of low BMI bodies. It is important to identify whether adaptation is exposure induced or an effect of a shift in attentional bias, because while the timeframe of the body size aftereffect is currently unknown, adaptation effects can decay quickly without re-exposure to adaptation stimuli [76]. A shift in attentional bias could prolong adaptation effects on body size estimation, because after the training participants are directing more attention to (and thus still adapting to) a particular body size.

In contrast to the low BMI attention training group, participants trained to attend to high BMI bodies showed no body size aftereffects. This finding is surprising, because body size adaptation studies on non-clinical populations consistently find participants exhibit body size aftereffects to both low and high BMI bodies [24–27,31]. While imbalanced aftereffects are uncommon in studies on non-clinical populations, a study on women with anorexia nervosa and bulimia nervosa found that participants adapted to high BMI bodies but not low BMI bodies [77]. The authors suggested that participants may have been preadapted to low BMI bodies due to a pre-existing attentional bias to low BMI bodies, and therefore measurable body size aftereffects could not be induced in a laboratory experiment. In our study, participants trained to attend to high BMI bodies did have an average negative attentional bias score at pre-training, indicating a possible pre-existing attentional bias to high BMI bodies. However, we are cautious about making inferences from the attentional bias scores given that they demonstrated unacceptably poor internal consistency. As this is the first study to use this novel training visual search task and the training was successful at adapting participants to low BMI bodies, future research is justified to explore whether modifications to the task increase the likelihood of aftereffects to high BMI bodies, especially because high BMI bodies are our target for therapeutic intervention. Possible modifications could include reducing the number of breaks and increasing the number of training trials and sessions to reduce the chance of adaptation decay. Alternatively, it is possible that training participants to search for non-body target stimuli (e.g. bars of specific orientations) has the unintended consequence of training participants to avoid body stimuli, and so visual search attention training tasks that require participants to search for target body sizes rather than adjacent non-body stimuli may be more effective at training attention.

## 5. Conclusion

We used a novel attention training task and found that young adult women trained to attend to low BMI bodies showed a body size aftereffect, i.e. they overestimated the size of subsequently presented body stimuli and thus selected a lower BMI body as ‘normal’ sized. Contrary to our expectations, the attention training did not induce adaptation to high BMI bodies and had no measurable effect on reaction times or body dissatisfaction. However, given the training was effective at inducing adaptation to low BMI bodies, modifications to the task (e.g. reducing the number of breaks and increasing the number of training trials and sessions) could make the task more effective at inducing an aftereffect following attention to high BMI bodies. The visual search task demonstrated unacceptably low internal consistency as a measure of attentional bias to body size, and therefore we recommend researchers explore other options (e.g. eye-tracking or ERPs) when assessing the effects of attention training on attention.

## Data Availability

The data for this experiment are publicly available via Open Science Framework: https://doi.org/10.17605/OSF.IO/4ZJRK (https://osf.io/4zjrk/) [78]. The data are provided in electronic supplementary material [79].
